# Timescale of hole closure during plasma membrane repair estimated by calcium imaging and numerical modeling

**DOI:** 10.1038/s41598-021-82926-6

**Published:** 2021-02-19

**Authors:** Martin Berg Klenow, Anne Sofie Busk Heitmann, Jesper Nylandsted, Adam Cohen Simonsen

**Affiliations:** 1 Department of Physics Chemistry and Pharmacy (FKF), Odense, Denmark; 2 University of Southern Denmark (SDU), Campusvej 55, 5230 Odense, Denmark; 3 Danish Cancer Society Research Center, Strandboulevarden 49, 2100 Copenhagen, Denmark; 4 Department of Cellular and Molecular Medicine, Faculty of Health Sciences, University of Copenhagen, Blegdamsvej 3C, 2200 Copenhagen, Denmark

**Keywords:** Biophysics, Systems biology

## Abstract

Plasma membrane repair is essential for eukaryotic cell life and is triggered by the influx of calcium through membrane wounds. Repair consists of sequential steps, with closure of the membrane hole being the key event that allows the cell to recover, thus identifying the kinetics of hole closure as important for clarifying repair mechanisms and as a quantitative handle on repair efficiency. We implement calcium imaging in MCF7 breast carcinoma cells subject to laser damage, coupled with a model describing the spatio-temporal calcium distribution. The model identifies the time point of hole closure as the time of maximum calcium signal. Analysis of cell data estimates the closure time as: $\langle t_c \rangle =5.45\pm 2.25$ s and $\langle t_c \rangle =6.81\pm 4.69$ s using GCaMP6s-CAAX and GCaMP6s probes respectively. The timescale was confirmed by independent time-lapse imaging of a hole during sealing. Moreover, the analysis estimates the characteristic time scale of calcium removal, the penetration depth of the calcium wave and the diffusion coefficient. Probing of hole closure times emerges as a strong universal tool for quantification of plasma membrane repair

## Introduction

The integrity of the plasma membrane of eucaryotic cells is required for maintaining cell homeostasis. During the life cycle of healthy organisms, the plasma membrane can undergo damage and rupture which may for example be inflicted by mechanical forces from the surrounding tissue, e.g. in muscles or in the lungs, or membrane holes can be induced by pore forming toxins produced by pathogens^1^. Metastatic cancer cells in particular, are subject to damage during their invasion into the extracellular matrix and exhibit specialized repair features involving S100 proteins^2^. In all cases, membrane holes must be sealed rapidly to secure cell survival and robust repair mechanisms have been evolutionary developed that involve the concerted action of a range of repair proteins, membrane fusion events and changes to the actin cytoskeleton^3–5^. The molecular repair mechanisms and the precise nature of membrane remodeling during repair are not fully understood, but subject to increasing interest in studies that often require an interdisciplinary approach^6^.

Plasma membrane repair is well known to be triggered by the influx of calcium at the damage site^7,8^ activating calcium-dependent proteins, membrane fusion events and cytoskeletal changes. Interestingly, it was recently shown that calcium impacts the pore lifetime in a lipid-only GUV system^9^ and such purely physical effects may also play a role in cell membrane repair. Pore lifetimes in GUVs subject to poration by localized plasmonic heating was previously estimated using the release of entrapped calcein^10^. In eucaryotic cells the extracellular calcium concentration is typically in the mM range whereas the intracellular concentration is in the low to mid nM range^11,12^. This $\times$10,000 fold concentration difference drives the calcium influx through membrane holes by passive diffusion.

An important example of repair proteins is the Annexin family^13^, many of which play a functional role during plasma membrane repair^14–16^. Annexins bind to negatively charged lipids in the presence of calcium and localize at the damage site during repair. Annexins can induce membrane fusion^17^, can form ordered 2D arrays on model membranes^18,19^ and induce negative membrane curvature of importance for the repair mechanisms^20–22^.

Plasma membrane repair can be viewed as occurring in several stages, each of which are important to identify when referring to the overall process of repair. Following the initial calcium influx and protein activation, the actual sealing of the hole has highest priority and can be expected to occur as early as possible in the repair sequence. Subsequent cleanup and recovery processes are important for returning the cell to its normal state, but these later events may in cell studies be challenging to discriminate from the initial repair response that is responsible for the actual hole sealing. Identifying the precise timescale of hole sealing during repair is therefore a crucial piece of information that marks the transition between distinct stages of repair and in particular separates the process of hole closure from subsequent events in the repair sequence.

A so-called resealing time in sea urchin eggs after micropipette injury with unspecified hole size, was previously estimated to around 10–30 s^23^ and later the same approach was applied to repair of fibroblasts^24^. Holes of $40 \times 10$ $\upmu {\mathrm{m}}^2$ in sea urchin eggs where demonstrated to seal within a few seconds in a micropipette study^25^. Repair in Drosophilia embryos after laser damage with varying hole sizes was studied by imaging and the repair time estimated to hundreds of seconds^26^. Repair of muscle fiber cells after exercise was studied using FM 1-43 staining of endo-membranes which stabilizes after 1-5 min, as corresponding to completion of repair^27^. In the above studies, the timepoint of hole closure during the repair sequence was not specifically addressed or defined. In fact, it appears that the concept of a repair/resealing time has not been consistently defined in the literature which makes comparison between studies more difficult. Here we propose to use the time of hole sealing as a clearly defined marker of the repair time.

A critical factor governing the spatiotemporal calcium distribution during damage/repair is the removal of calcium entering through the wound. Many cellular functions depend on precise calcium levels in the cytosol and several transport systems are regulating the concentration. In resting cells, the low calcium level is mainly regulated by the plasma membrane $\hbox {Ca}^{\mathrm{2+}}$transport ATPase (PMCA) and the $\hbox {Na}^+$/$\hbox {Ca}^{\mathrm{2+}}$exchanger (NCX). When the calcium level is increased as during plasma membrane damage, regulation is supplemented by the sarcoendoplasmic reticulum $\hbox {Ca}^{\mathrm{2+}}$-ATPase (SERCA) and the mitochondrial $\hbox {Ca}^{\mathrm{2+}}$uniporter (MCU)^28,29^. Moreover, calcium-binding proteins such as annexins may transiently fixate calcium during repair. In the simplest possible model of repair, the calcium level is determined by (1) influx through the membrane wound until closure and (2) removal of calcium by pumps or by binding to proteins.

Here we are focusing on quantification of the timescale of hole closure in repair of MCF7 breast carcinoma cells. Confocal imaging of intracellular calcium using the GCaMP6s probe in both a cytosolic^30^ and membrane-bound^31^ form shows the spatio-temporal calcium distribution after UV-laser damage. To support interpretation of the cell data, we establish a theoretical model for the calcium distribution, taking into account diffusion through the membrane wound, hole closure and removal of calcium from the cytosol. The model identifies the time of maximum intracellular calcium as the time point of hole closure, independent from other parameters in the model. The results demonstrate that the timescale of hole closure can be simply and accurately derived from calcium imaging data. The approach will be helpful to identify or eliminate potential candidates of repair mechanisms based on timescales.

## Methods and materials

### Cell culture and expression of the calcium sensors

MCF7 breast cancer cells (ATCC no.: HTB-22) from human breast carcinoma were cultured in Gibco RPMI medium with 6 % FCS (fetal calf serum) and antibiotics and kept in a 37$^{\circ }$C $\text {CO}_2$ incubator. Expression plasmids containing the calcium indicators GCaMP6s (cytosolic probe) and GCaMP6s-CAAX (plasma membrane targeted probe) were used. Specifically, pGP-CMV-GCaMP6s was a gift from Douglas Kim & GENIE Project (Addgene plasmid # 40753; http://n2t.net/addgene:40753; RRID:Addgene_40753)^30^ while pGP-CMV-GCaMP6s-CAAX was a gift from Tobias Meyer (Addgene plasmid #52228; http://n2t.net/addgene:52228; RRID:Addgene_52228)^31^. The MCF7 cells were transfected with one of the calcium sensor plasmids. Transfection was accomplished by using Lipofectamine LTX Transfection Reagent (Invitrogen). Imaging was done in MatTek 35 mm glass bottom dishes with 45.000 cells seeded in a liquid volume of 1.5 mL. Before imaging 25 mM Gibco HEPES (4-(2-hydroxyethyl)-1-piperazineethanesulfonic acid) was added to the cell medium to provide an enhanced buffering capacity for the cell culture outside the $\text {CO}_2$ incubator.

### UV laser-induced membrane damage

For induction of membrane damage, MCF7 breast carcinoma cells were injured by a 355 nm pulsed UV-laser (Rapp OptoElectronic) at 37 $^\circ$C. Cells were injured by irradiating a small region (1–2 $\upmu {\mathrm{m}}^2$) for < 10–100 ms with the following settings: 2.6% power, 200 Hz repetition rate, pulse energy > 60 $\upmu$J, pulse length < 4 ns.

### Spinning disc fluorescence microscopy

Images were acquired through a 63X objective with the inverted microscope Eclipse Ti-E (Nikon) paired with UltraVIEW VoX Spinning Disk (PerkinElmer). Control of hardware as well as intensity measurements were performed with Volocity software (PerkinElmer). Imaging was performed in the GFP-channel at 488 nm excitation for both of the calcium sensors. Cells were imaged for approximately 2 minutes with a frame rate of 2 frames per second including around 20 frames before the injury was induced.

### Image analysis for quantification of the calcium probe intensities

For image analysis of the time-lapse spinning disc fluorescence data a MATLAB script was developed. The script detects the cell from the background by using a global image threshold. The location of the cell injury is defined by the user along with a background region for correction of intensities. For each image in the time-lapse sequence, the intracellular mean intensity is computed within the boundary of the cell in the image. For computation of the radial intensity distribution, circular bands with centers at the injury site are defined and the mean intensity within each of the bands is calculated. Thus, for each time point a radial intensity distribution is obtained. The background distribution before damage was subtracted such that the final radial distribution *I*(*R*, *t*) only reflects increased levels of calcium. The intensity decays to zero when calcium is cleared from the cell allowing a time-averaged sum to be computed:

1 $$\begin{aligned} I(R)=\int I(R,t) dt \end{aligned}$$

The corresponding normalized radial probability density *P*(*R*) is:

2 $$\begin{aligned} P(R)=\frac{I(R)}{\int I(R) dR} \end{aligned}$$

The expectation value *E*(*R*) of the distribution *P*(*R*) is the penetration depth of the calcium wave which provides an estimate of a characteristic depth into the cytosol reached by the calcium ions after damage.

3 $$\begin{aligned} E(R)=\int R P(R) dR \end{aligned}$$

and the corresponding standard deviation $\sigma (R)=\sqrt{E(R^2)-E(R)^2}$ is an estimate of the width of the calcium wave. The total calcium signal was determined within the footprint of the cell. We note that all recorded intensity maxima were sharp and without any sign of a plateau, indicating that the calcium probe was not saturated under the conditions of the experiment.

**Figure 1:**
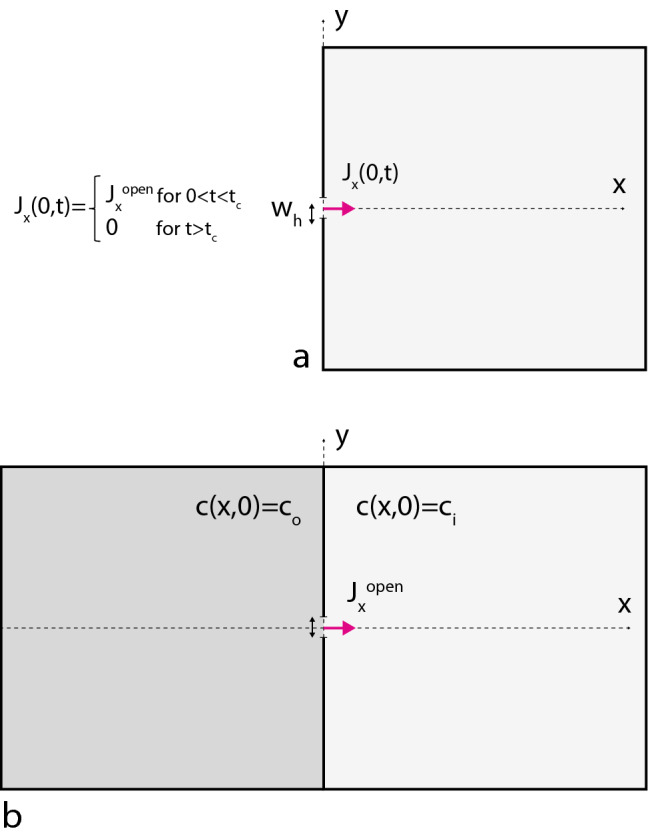
Box model for a cell containing a transient plasma membrane hole. Model in (**a**) shows the full model including hole closure at time $t=t_c$. Model in (**b**) is a 2-box model for the cell exterior/interior with an always-open hole, used for determining the time-dependent influx $J_x^{\mathrm{open}}(t)$ through the hole.

**Figure 2:**
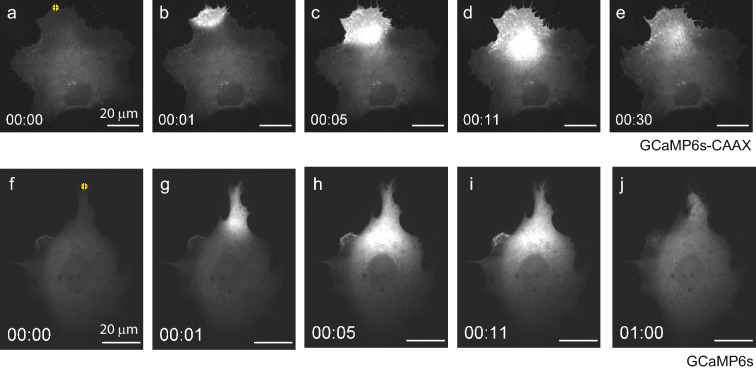
Time-lapse fluorescence data for the cytosolic calcium distribution in an MCF7 breast carcinoma cell during UV-laser induced plasma membrane damage and repair. Sequence in (**a**–**e**) was acquired with the membrane bound probe GCaMP6s-CAAX while sequence in (**f**–**j**) was acquired with the cytoplasma probe GCaMP6s. Laser damage to the plasma membrane was induced at the marked point (yellow) in the cell at time t = 0 s. All images within one sequence are displayed at the same intensity scale.

## Model of the calcium distribution

A numerical model for calcium transport into a cell through a membrane hole was established. The model is based on the 2D box-geometry shown in Fig. 1a where the cell interior is described by a square box with a sufficient size that the flux of calcium through the hole does not saturate on the timescale of relevance for repair. The time and space-dependent calcium concentration inside the cell *c*(*r*, *t*) is approximately governed by the diffusion equation with a diffusion coefficient D of calcium in the cytoplasm:

4 $$\begin{aligned} \frac{\partial c}{\partial t}=D \nabla ^2 c \end{aligned}$$

The removal of excess calcium from the cell interior is modeled as a constant negative source term $-K$ in the diffusion equation, such that calcium is removed at a constant rate. The pumping term is set to zero at positions in the cell where the concentration *c* has fallen to the normal cytosol concentration $c_i$:

5 $$\begin{aligned} \frac{\partial c}{\partial t}|_{\mathrm{pump}}= {\left\{ \begin{array}{ll} -K &{} c_i<c \\ 0 &{} c<c_i \end{array}\right. } \end{aligned}$$

The final equation governing the calcium concentration is thus:

6 $$\begin{aligned} \frac{\partial c}{\partial t}=D \nabla ^2 c +\frac{\partial c}{\partial t}|_{\mathrm{pump}} \end{aligned}$$

As a model for a membrane hole, a segment of the box of width $w_h$ will be transiently open in the time interval t = [0, $t_c$]. The influx of calcium ions through the hole is implemented as a Neumann boundary condition over the width of the hole, specifying the time-dependent influx $J_x(0,t)=J_x(t)$ of calcium through the hole. The closure of the membrane hole via plasma membrane repair is modeled as a step function in the influx:

7 $$\begin{aligned} -D\frac{\partial c}{\partial x}|_{x=0}=J_x= {\left\{ \begin{array}{ll} J_x^{\mathrm{open}}(t) &{} 0<t<t_c \\ 0 &{} t>t_c \end{array}\right. } \end{aligned}$$

where $J_x(t)$ is the time dependent flux through the membrane hole. To determine the flux $J_x^{\mathrm{open}}$ in the open state, a 2-box model describing both the cell exterior *and* the cell interior is implemented as shown in Fig. 1b. The 2-box model has the hole always in the open state, but the model is useful for accurately determining the flux through the hole, to be used in the 1-box system that includes hole closure. The initial condition for the 2-box system is:

8 $$\begin{aligned} c(x,0)= {\left\{ \begin{array}{ll} c_o &{} x<0 \\ c_i &{} x>0 \end{array}\right. } \end{aligned}$$

where we use the following values: $c_o$ = 2 mM, $c_i$ = 100 nM. From the solution to the 2-box system, the influx $J_x^{\mathrm{open}}(t)$ at the position of the hole is calculated and fitted to a power law function: $J_x^{\mathrm{open}}(t) \propto t^{-\beta }$ and subsequently used in Eq. (7).

The walls of the box outside the hole are described by Neumann boundary conditions with zero concentration gradient, describing zero transport across the walls of the box outside the hole, i.e. a non-leaking plasma membrane.

Equation (6) was numerically solved in MATLAB using the Partial Differential Equation (pde) Toolbox with custom-written m-functions. A maximum mesh length of 1 $\upmu$m was used for the discretization of the simulation box.

## Results

The distribution of intracellular calcium following plasma membrane damage and repair is studied by two approaches: (1) Fluorescence based imaging of calcium in MCF7 cells and (2) a computational model for the calcium distribution used to simulate membrane rupture and repair. The intracellular calcium in MCF7 cells following rapid UV-laser induced damage to the plasma membrane was monitored with the cytosolic probe GCaMP6s^30^ or the membrane-bound probe GCaMP6s-CAAX^31^.

### Timescale of hole closure estimated from the total calcium intensity

We first examine qualitatively the spatio-temporal intensity distribution of the calcium probe after membrane damage. The plasma membrane is ruptured by UV-laser irradiation at a localized point selected close to the border of the cell. This was chosen to minimize injury of intracellular organelles which may otherwise compromise cell survival. The emission intensity of the GCaMP6s calcium probe is low at the normal background $\hbox {Ca}^{\mathrm{2+}}$concentration ($\sim$100 nM) of resting cells. Rupture triggers the influx of extracellular $\hbox {Ca}^{\mathrm{2+}}$($\sim$2 mM) as driven by the concentration gradient until the plasma membrane hole is sealed. The response of the membrane-bound probe GCaMP6s-CAAX in Fig. 2a–e, shows a high emission intensity localized near the damage for short times after rupture. Initially, the $\hbox {Ca}^{\mathrm{2+}}$signal propagates rapidly into the cell followed by a slowing down of propagation and decrease in overall intensity.

Rupture will induce a flux in and out of the cell of molecules, depending on their concentration gradient. A membrane bound probe such as GCaMP6s-CAAX, is expected to be the most reliable reporter of $\hbox {Ca}^{\mathrm{2+}}$entry because it is relatively immobile. As a reference, we made parallel measurements using the cytosolic probe GCaMP6s shown in Fig. 2f–j. The intensity distribution shows qualitatively the same behavior as the membrane bound probe, but with a slightly lower signal to noise ratio.

**Figure 3:**
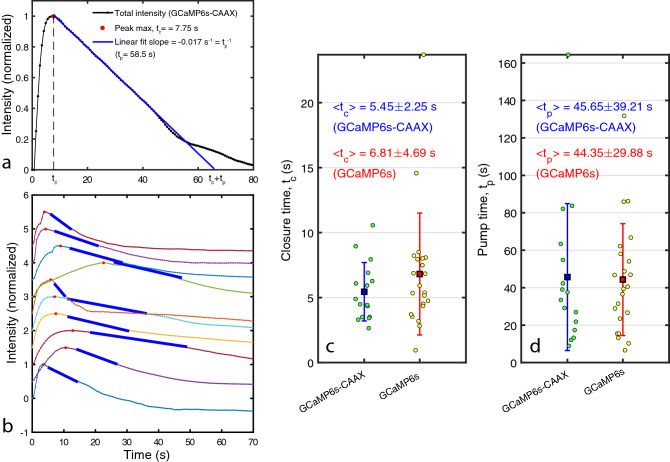
Plot of the calcium-probe (GCaMP6s-CAAX) intensity within the footprint of an MCF7 cell during laser damage and repair (**a**). Intensity is normalized to the maximum intensity reached. The peak maximum at time $t=t_c$ is indicated (red dot) and a linear fit (blue line) to the intensity decrease during repair. The pump time $t_p$ is defined as the inverse of the slope of the linear fit. Examples of 10 random intensity curves (probe: GCaMP6s-CAAX) with associated peak maxima and linear fits to the decay region (**b**). Curves in (**b**) are offset vertically for clarity. The distribution of measured closure times $t_c$ and the mean values for the GCaMP6s-CAAX (N = 16) and the GCaMP6s (N = 22) probes (**c**). The distribution of the pump times $t_p$ and the mean values for the GCaMP6s-CAAX and the GCaMP6s probes (**d**). Uncertainties in (**c**,**d**) are ± one standard deviation.

**Figure 4:**
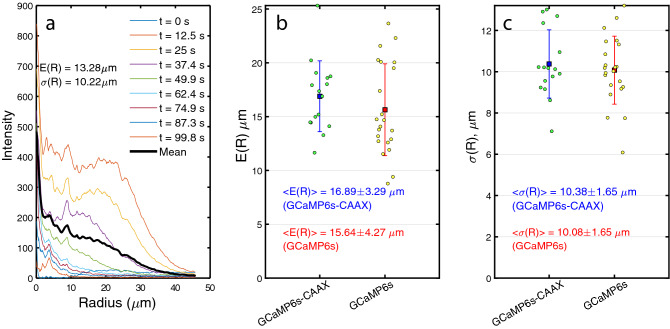
Analysis of the radial distribution of fluorescence intensities from the point of UV-laser damage. Example of a sequence of radial intensity distributions for the probe GCaMP6s-CAAX (**a**). The mean intensity distribution (solid black) is the time average of the radial intensities. The expectation value of the radius, using the mean intensity distribution, is E(R) and is a measure of the characteristic penetration depth of calcium into the cell after damage. The corresponding standard deviation of the mean radial distribution is $\sigma$(R). The scatter and mean value of E(R) for a number of cells labeled with the probes GCaMP6s-CAAX (N = 16) and GCaMP6s (N = 22), respectively (**b**). The corresponding scatter and mean value of the standard deviation $\sigma$(R) for the same cells (C). Uncertainties in (**b**,**c**) are ± one standard deviation.

The mean intensity of the calcium probe was quantified within the footprint of the cell and normalized to one for the maximum intensity reached. The background level before damage and after completion of repair was subtracted, bringing the base level to zero. A typical intensity curve in Fig. 3a shows a rapid initial increase to a maximum followed by a slower decrease. The time $t=t_c$ of maximum calcium probe intensity is the approximate time where the intracellular calcium concentration due to rupture is maximal. We determine the position of the maximum by fitting a parabola in the intensity range 1.00-0.95 (i.e. upper 5 %). In a simple description, the calcium level is set by a competition between entry through the membrane hole and calcium removal by cellular processes such as pumps. We therefore denote $t_c$ the *closure time* as an estimate of the time point where the hole is sealed. This interpretation is further substantiated by modeling, discussed below.

The decrease in intensity in Fig. 3a follows approximately a linear trend for times shortly after the maximum. A linear fit to the intensity after the maximum was performed for intensities in the interval of 0.5-0.95. If the decrease was perfectly linear for times after the maximum, the inverse of the slope would be the *pump time* $t_p$ taken to restore calcium to the normal level in the cell. Although the actual pump time in our cells is not identical to $t_p$, it is a convenient quantification of a characteristic timescale for calcium clearance after repair. A random sample of 10 intensity curves are shown in Fig. 3b, indicating the level of variation among cells.

The distribution of $t_c$ and $t_p$ is plotted in Fig. 3c,d. The mean closure times for the two calcium probes are: $\langle t_c \rangle =5.45\pm 2.25$ s (GCaMP6s-CAAX, N = 16 cells) and $\langle t_c \rangle =6.81\pm 4.69$ s (GCaMP6s, N = 22 cells) which are not statistically different. The variation originates mainly from structural and compositional variations between the cells and the size and location of the membrane hole and to a smaller extent from measurement uncertainty. Further characterization of these factors in single cells could help locating the origin of the variation in $t_c$ between cells. The mean pump time determined for the two calcium probes are: $\langle t_p \rangle =45.65 \pm 39.21$ s (GCaMP6s-CAAX, N = 16 cells) and $\langle t_p \rangle = 44.35 \pm 29.88$ s (GCaMP6s, N = 22 cells) which are also not statistically different. It is a significant finding that the mean timescale for recovery of the calcium concentration is approximately 8-10X longer than the mean timescale for sealing of the hole. Thus, it appears that the immediate sealing of the plasma membrane hole has priority over the subsequent recovery of the cell homeostasis to the state of an un-injured cell.

### Radial distribution of calcium yields the penetration depth of the calcium wave

In addition to tracking the total probe intensity within the cell, we also consider the spatio-temporal distribution of the calcium probe relative to the point of membrane damage. This gives important information about the length scale that calcium travels into the cytosol after damage. Using image analysis, the emission intensity of the calcium probe was averaged over regions with equal distance to the point of damage thereby creating a radial intensity distribution. Figure 4a shows a typical time sequence of radial distributions acquired with the membrane probe GCaMP6s-CAAX. The distribution being initially close to zero is rapidly increased near the damage site after UV-injury and then decays back to the baseline level as the original calcium concentration is restored. The detailed shape of the radial distribution varies between experiments, reflecting variations in topography and footprint between individual cells. The time average of the radial intensity distribution (solid black) can conveniently be used to estimate the characteristic penetration depth of the calcium wave into the cytosol and its spread, via calculation of the expectation value *E*(*R*) and the standard deviation $\sigma (R)$ of the distribution, respectively (see Methods). Figure 4b shows the scatter of the calcium penetration depth *E*(*R*) and its mean value $\langle E(R) \rangle$ determined from the radial intensity distribution of the calcium probes GCaMP6s-CAAX and GCaMP6s.

The two probes result in average penetration depths of $\langle E(R)\rangle = 16.89 \pm 3.29$ $\upmu$m (GCaMP6s-CAAX, N = 16) and $\langle E(R)\rangle = 15.64 \pm 4.27$ $\upmu$m (GCaMP6s, N = 22). The corresponding standard deviations for the two probes are shown in Fig. 4c and give the values $\langle \sigma (R)\rangle =10.38 \pm 1.65$ $\upmu$m (GCaMP6s-CAAX) and $\langle \sigma (R)\rangle =10.08 \pm 1.65$ $\upmu$m (GCaMP6s).

The standard deviations provide a characteristic length scale for the spread of the calcium wave in the cytosol during damage and repair and therefore a measure of the degree of localization of calcium. Both the penetration depth and the spread of the calcium wave, as estimated above, produce consistent numbers for the two calcium probes. The same level of consistency between the probes was found for the analysis of the mean intensities in Fig. 3. This indicates that the spatio- temporal distribution of calcium during damage and repair can reliably be estimated and is not significantly influenced by the probe being membrane-bound or not.

### Numerical modeling validates the analysis of calcium data

In order to validate and understand the experimental calcium distributions in cells we decided to model the spatio-temporal distribution of calcium using a numerical diffusion model as shown in Fig. 1. The purpose is to examine if a simplistic model of the calcium influx, containing a minimal number of parameters, can reproduce the essential features of the experimental calcium distributions in the cytosol. Most importantly we seek to establish a connection between the intracellular calcium distribution and the timescale of hole closure which appears explicitly in the underlying model.

The 2D model approximates the cell interior as a square box of sufficient size that the influx of calcium through the hole does not saturate appreciably on the timescale of relevance for repair. In the experiments, the surface adhering MCF7 cells are indeed flattened and have approximately a 2D shape near the site of injury at the cell boundary. The plasma membrane hole is modeled as being open for times $0<t<t_c$ and closed for times $t>t_c$. Intracellular removal of calcium during repair is described in the model by a constant, uniform pumping rate *K* in Eq. (5). Thus, the model approximately describes the spatio-temporal calcium distribution in the cell close to a hole, but without including details of the cell shape.

**Figure 5:**
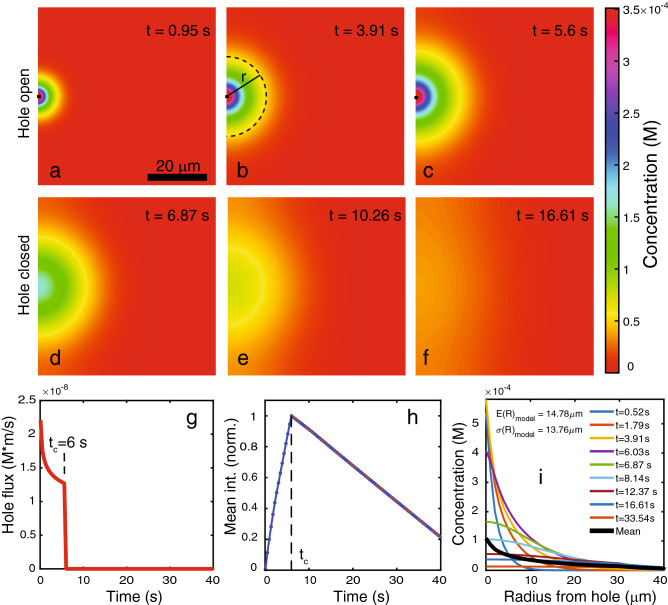
Output from model of the intracellular calcium distribution. Spatial distribution of the calcium concentration (unit = M) at selected time points before and after hole closure at time $t_c$ = 6 s (**a**–**f**). Diffusion coefficient $D=2.7\cdot 10^{-11} \, {\mathrm{m}}^2/\mathrm{s}$, hole width $w_h=1 \, \upmu {\mathrm{m}}$, initial extracellular calcium conc. $c_o=2$ mM, initial intracellular calcium conc. $c_i=100$ nM, pump rate $K=5 \cdot 10^{-7} \, {\mathrm{m}}/\mathrm{s}$. Time dependence of the hole flux $J_x$ as determined from the solution to the 2-box model in figure 1b (**g**). Mean concentration inside simulation box normalized to the peak maximum (**h**). Radial distribution of the calcium concentration relative to the membrane hole at selected time points (**i**).

**Figure 6:**
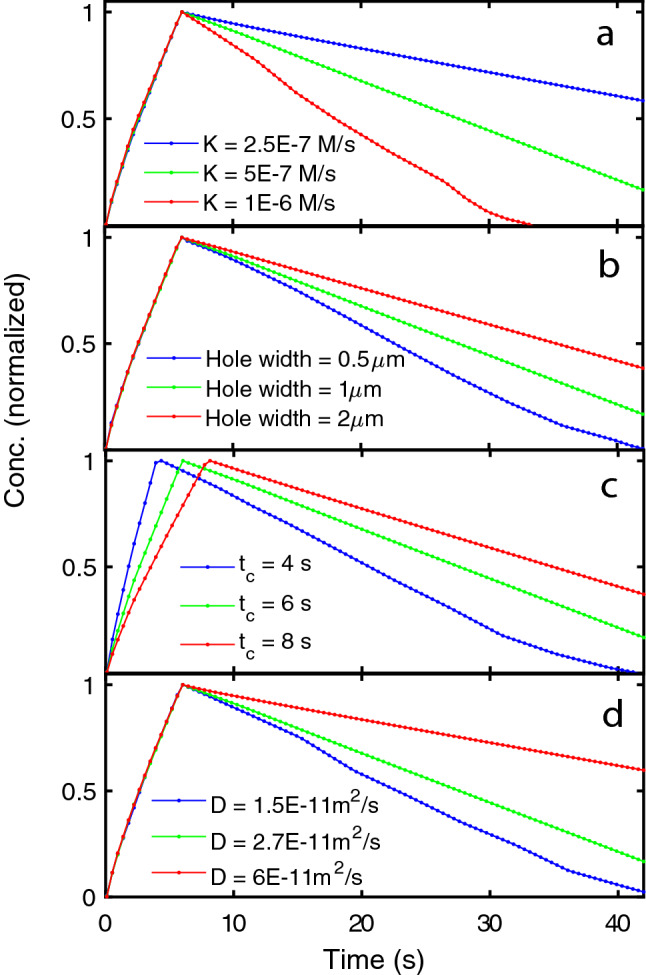
Variation of the mean calcium concentration in the simulation box upon variation of selected parameters in the model (**a**–**d**). The values of the varied parameter are indicated in the legends while the non-varied parameters have the values used in Fig. 5. The time dependence of the mean concentration was in all cases normalized to the peak maximum. It is noted that variation of the pump rate *K* (**a**), the hole width $w_h$ (**b**) and the diffusion coefficient *D* (**d**) affect only the slope of decay after $t_c$. Variation of the hole closure time $t_c$ (**c**) affects both the position of the peak maximum and the decay slope. The peak maximum is identical to $t_c$ within the numerical resolution.

**Figure 7:**
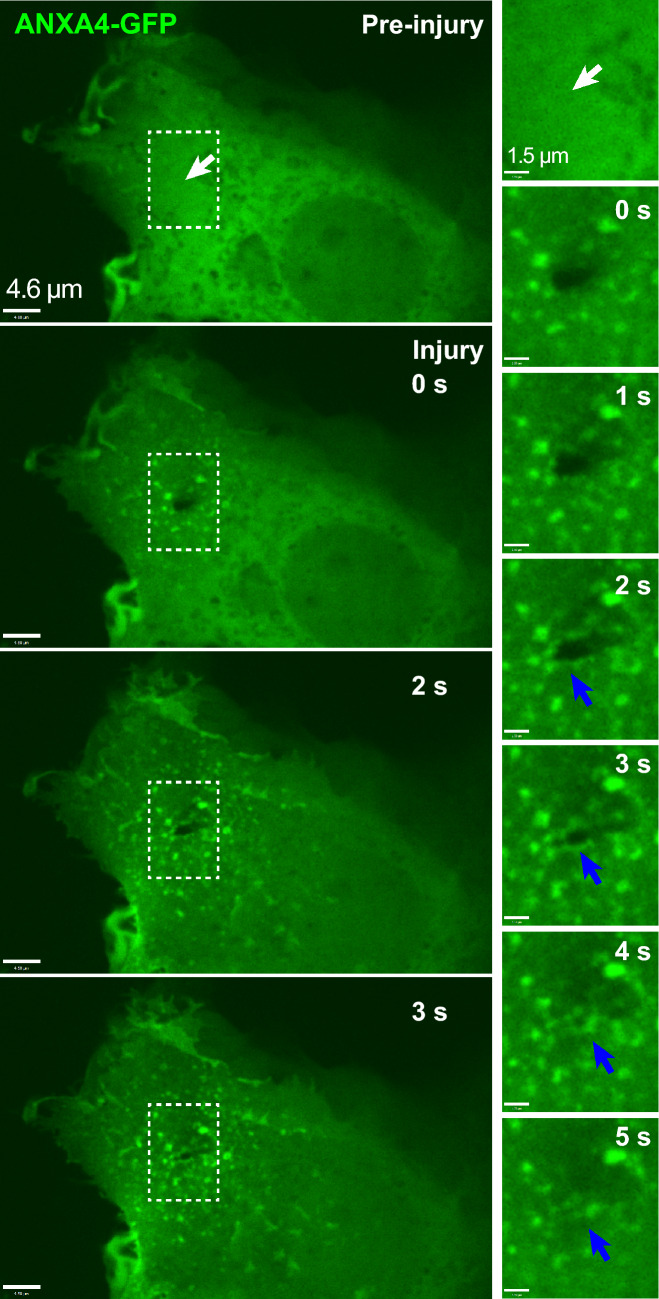
Sequential images of MCF7 cell expressing ANXA4-GFP and exposed to laser injury by shooting through the plasma membrane by laser at the injury site (white arrow). A zoom of the injury region shows that ANXA4-GFP localizes to wound edges (blue arrow) followed by closure of the membrane hole within 5 s after damage.

Figure 5a–f shows the calcium distribution generated by the model using the parameter values indicated. A set of parameter values for the model where chosen, that produce a calcium distribution approximating the average result extracted from cell experiments. Specifically the values of the initial calcium concentrations $c_o=2$ mM, $c_i=100$ nM where fixed as where the closure time $t_c=$ 6 s. The hole width $w_h$ and the pump rate *K* are strongly coupled and both influence the slope after the maximum. Therefore the hole width was set to $w_h=1 \, \upmu {\mathrm{m}}$ as an estimate of the average value in the cell experiments. The pump rate was estimated to $K=5 \cdot 10^{-7}$ M/s to conform with the average slope in the cell experiments. The diffusion coefficient *D* mainly influences the penetration depth into the cytosol and was estimated to $D=2.7\cdot 10^{-11} \, {\mathrm{m}}^2/\mathrm{s}$ to obtain agreement with the penetration depth in the cell experiments.

The calcium distribution in Fig. 5a–c correspond to time points before hole closure ($t_c=$ 6 s) and show a circular penetration of calcium into the cytosol due to influx through the hole as plotted in Fig. 5g. After hole closure at $t=t_c$, the calcium front stops expanding in Fig. 5d–f and the calcium concentration decays to the background level.

The mean concentration in the simulation box, normalized to the maximum, is shown in Fig. 5h as function of time. It displays an increase to a maximum reached at time $t=t_c$ followed by a linear decay, as expected from the constant pumping rate in the numerical model. The curve shape agrees qualitatively with the experimental intensity in Fig. 3a, albeit with the difference that the experimental intensity has a softer maximum, most likely because hole closure in a cell occurs gradually rather than discretely as in the model. However, the important point of Fig. 5h is that the time point of maximal intensity coincides with the time point of hole closure $t_c$. This result of the modeling provides an argument for estimating the timescale of hole closure in the cell experiments as the position of the maximum in the mean intensity, as was done in Fig. 3c.

The radial distribution of the calcium concentration relative to the center of the hole is displayed in Fig. 5i for selected time points. Comparing with the radial distribution in the cell experiments in Fig. 4a, the model also displays accumulation of calcium near the damage site and a decay to the background after hole closure. Moreover, from the time-average of the radial distributions (black curve), the penetration depth $E(R)=14.78 \, \upmu {\mathrm{m}}$ and the spread of the calcium wave $\sigma (R)=13.76 \, \upmu {\mathrm{m}}$ in the model was calculated. The penetration depth from the model is within the range of results from cell experiments whereas the spread in the model is slightly higher. It demonstrates that despite the simplicity of the 2D model and the lack of description of the detailed cell shape, semi-quantitative agreement with the spatial distribution of the calcium wave in cells can be obtained.

The time dependence of the mean concentration is further examined in Fig. 6a–d when varying selected parameters in the model. Variation of the hole width $w_h$, the closure time $t_c$ or the diffusion coefficient *D* all result in changes to the slope after the maximum, because these parameters influence the amount of calcium entering the cell through the hole. Obviously, the pump rate *K* influences the slope after the maximum by modifying the rate of calcium removal after hole closure. Importantly, the position of the maximum is only influenced by variation of the hole closure time $t_c$ in the model and not by other parameters; again supporting that the time point of maximum calcium intensity can be used as a reliable estimate of the hole closure time.

### Confocal imaging of a hole during repair confirms the timescale of hole closure

To substantiate the quantification of hole closure dynamics obtained by calcium imaging, we performed a direct visualization, using spinning disc confocal microscopy, of hole closure in MCF7 cells after UV-laser damage, as previously implemented^20^. Figure 7 shows MCF7 cells expressing Annexin A4 GFP (ANXA4-GFP) with the plasma membrane hole being clearly resolved at the time points after injury. A zoom of the region of membrane damage shows the gradual shrinking of the membrane hole at time points 0-4 s and eventual disappearance (within resolution) of the hole after 5 s. Interestingly, this timescale of hole closure obtained by imaging, is fully compatible with the closure times $t_c$ in Fig. 3c estimated from calcium imaging. This indicates that optical microscopy of hole closure corresponds to actual sealing of the hole and supports that calcium imaging is useful for estimating closure times.

## Discussion

The results presented here demonstrate that unambiguous quantification of the timescale of hole closure during membrane repair is feasible. After injury, the mean calcium signal from a cell increases to a maximum followed by a decrease to the base level in qualitative agreement with calcium data first obtained by Steinhardt et al.^23,24^. However, in these earlier studies the ’resealing’ time was taken as the time when the calcium level had returned to stationary after damage/repair which is not supported by physical model developed here. Similar calcium signals were also recently published in a study of annexin A6^32^. In these studies the calcium data required to estimate the time of hole closure were available, but not analyzed in the way done here. Proper interpretation of the calcium signal during repair is facilitated by validation with a physical model of calcium transport. The mean calcium level in the model reproduces the signal recorded in MCF7 cells and allows an examination of the role of individual model parameters. Specifically, the position of the intensity maximum coincides with the hole closure time and is unaffected by other variables in the model. Analysis of the spatial distribution of calcium during rupture and repair yielded a time-averaged characteristic penetration depth and spread of the calcium wave. The two calcium sensitive probes used in this study differ by being membrane bound (GCaMP6s-CAAX) or not (GCaMP6s). The spatio-temporal quantification of the calcium signal is not statistically different for the two probes, suggesting that also the non membrane-bound probe can be reliably used for quantification of the calcium distribution. Comparing with the model, the effective diffusion coefficient of calcium was estimated to $D=2.7\cdot 10^{-11} \, {\mathrm{m}}^2/\mathrm{s}$ = $27 \, \upmu {\mathrm{m}}^2/\mathrm{s}$. In comparison, the diffusion coefficient of free calcium in cytoplasm (Myxicola worms) was first estimated by Donahue et al. to $D=530 \, \upmu {\mathrm{m}}^2/\mathrm{s}$^33^. Revised measurements by the same group later gave results in the range of 10–$50 \, \upmu {\mathrm{m}}^2/\mathrm{s}$^34^ in agreement with the our estimate. Apparent calcium diffusion coefficients were estimated by Straube et al. in stochastic simulations including molecular crowding and buffer effects. Results were in the range of 20–$40 \, \upmu {\mathrm{m}}^2/\mathrm{s}$ for mobile or immobile buffers with low affinity^35^ also in full agreement with our estimate.

The model for the calcium distribution demonstrates that a minimal 2D description reproduces the essential features in the experimental data obtained in cells, suggesting that the underlying dynamics during repair has been captured. The good quantitative performance of the model may partly be ascribed to the flat geometry of the cells near their border approximating a 2D system. A more accurate description of the individual cell shape would be expected to further improve the quantitative agreement. The shape of the intensity maximum is sharp in the model whereas it is rounded in the experimental data. This difference is due to the discrete on/off description of hole closure in the model whereas in reality the hole shrinks gradually before closure. The model could therefore be refined to include a gradual and continuous description of hole closure. This would allow us to establish a more precise relationship between the progress of hole closure and the calcium profile. Other refinements to the model include a non-constant pumping term and the incorporation of a mobile fluorescent probe that binds to calcium to produce the measured signal. But these refinements come at the expense of an increased number of parameters in the model.

The availability of accurate data for the timescale of hole closure is useful e.g. for comparison with potential models of membrane repair. Proposed repair mechanisms may take longer than what is compatible with the timescale of hole closure or oppositely, the timescale of closure may confirm the dynamics of the proposed repair mechanism. As an example, lipid diffusion in membranes will typically have $D\sim 1\, \upmu {\mathrm{m}}^2 /s$ giving a r.m.s diffusion distance over $t_c= 6$ s of $\sigma = \sqrt{4 D t_c} \sim 5 \,\upmu$m setting a limit to the radius from which lipids can be recruited to the injury. ANXA5 was shown to form 2D crystals on bilayers in buffer, within 5 s^19^ and this type of lattice formation has been implicated in repair^18^. Curvature and neck formation around membrane holes was proposed as the initial step in repair^20^. Membrane curvature and rolling induced by ANXA4 happens with a time scale of order 1 s, well within the limit of hole closure. In general, the short time scale of hole closure measured here, speaks in favor of a repair mechanism with components that are quickly activated and already located close to the plasma membrane before injury, such as annexins. In the light of the essential function of plasma membrane repair for cell life, accurate quantification of hole closure dynamics is important for identifying and refining models for repair.

## Supplementary Information

Supplementary Legends.

Supplementary Video 1.

Supplementary Video 2.

## Data Availability

The datasets generated and analysed during the current study are available from the corresponding author on reasonable request.
