# Supplementary material for: Timescale of hole closure during plasma membrane repair estimated by calcium imaging and numerical modeling
Source: Sci Rep. 2021 Feb 19;11:4226. doi: 10.1038/s41598-021-82926-6 (PMC7895973; doi:10.1038/s41598-021-82926-6)
Supplement: Supplementary file 1 — Supplementary Legends. [file 41598_2021_82926_MOESM1_ESM.pdf]

Supplementary material for:
Timescale of hole closure during plasma membrane repair estimated by calcium imaging and numerical modeling

Martin Berg Klenow[a,b], Anne Sofie Busk Heitmann[c], Jesper Nylandsted[c,d], Adam Cohen Simonsen[a,b,e]

[a]*Department of Physics Chemistry and Pharmacy (FKF)*
[b]*University of Southern Denmark (SDU)*
Campusvej 55, 5230 Odense M, Denmark
[c]*Danish Cancer Society Research Center*
Strandboulevarden 49, 2100 Copenhagen Ø, Denmark
[d]*Department of Cellular and Molecular Medicine, Faculty of Health Sciences*
University of Copenhagen, Blegdamsvej 3C, DK-2200 Copenhagen N, Denmark
[e]*Corresponding author (adam@sdu.dk)*

Supplementary video S1: Video showing the full time-lapse sequence corresponding to the frames in figure 2a-2e. Fluorescence data for the cytosolic calcium distribution in an MCF7 breast carcinoma cell during UV-laser induced plasma membrane damage and repair using the membrane bound probe GCaMP6s-CAAX. Scalebar is 20 μm.

Supplementary video S2: Video showing the full time-lapse sequence corresponding to the frames in figure 5a-5f. The xy-scale is length in m.
